# Supplementary material for: Biological Containment for African Swine Fever (ASF) Laboratories and Animal Facilities: The Italian Challenge in Bridging the Present Regulatory Gap and Enhancing Biosafety and Biosecurity Measures
Source: Animals (Basel). 2024 Jan 30;14(3):454. doi: 10.3390/ani14030454 (PMC10854939; doi:10.3390/ani14030454)
Supplement: Supplementary file 1 [file animals-14-00454-s001.zip › Table S1.pdf]

Table S1. Principles of biological containment appropriate for diagnostic laboratories

	Minimum requirements	Additional requirements
General environment	Normal atmospheric pressure.<br>Dedicated rooms limited to defined procedures.	Normal atmospheric pressure.<br>Dedicated rooms, used exclusively for classical swine fever or ASF diagnostic procedures.<br>Potentially contaminated waste treated to inactivate ASF virus (heat or chemical).
Laboratory clothing	Dedicated outer clothing used only in the ASF virus unit.<br>Disposable gloves for all manipulations of infected material.<br>Outer clothing sterilised before removal from unit, or washed at a high temperature within unit	Complete change of clothes on entry.<br>Laboratory clothing used only in the ASF virus unit. Disposable gloves for all manipulations of infected material.<br>Clothing sterilised before removal from unit, or washed at a high temperature within unit.
Control of personnel	Entry to unit limited to named, trained personnel. Wash and disinfect hands on leaving unit.<br>Personnel not permitted to visit premises with pigs for 48 hours after leaving unit	Entry to unit limited to named, trained personnel.<br>Wash and disinfect hands on leaving unit.<br>Personnel not permitted to visit premises with pigs for 48 hours after leaving unit.
Equipment	Biological safety cabinet (class I or II) used for all manipulations of live virus. Cabinet should have double HEPA filtration of exhaust air.<br>All equipment needed for laboratory procedures to be available within the dedicated laboratory suite.	
