# Supplementary material for: Biological Containment for African Swine Fever (ASF) Laboratories and Animal Facilities: The Italian Challenge in Bridging the Present Regulatory Gap and Enhancing Biosafety and Biosecurity Measures
Source: Animals (Basel). 2024 Jan 30;14(3):454. doi: 10.3390/ani14030454 (PMC10854939; doi:10.3390/ani14030454)
Supplement: Supplementary file 1 [file animals-14-00454-s001.zip › Table S2.pdf]

Table S2. Bio-safety requirements for experimental animal rooms

	**Requirements**
General environment	Negative-pressure-controlled ventilation. One HEPA filtration of exhaust air.<br>Facility for complete decontamination or fumigation at end of experiment.<br>All solid and liquid waste effluents treated to inactivate ASF virus (heat/incineration or chemical).
Laboratory clothing	Complete change of clothes on entry. Clothing sterilised before removal from unit, or washed at a high temperature within unit.
Control of personnel	Entry to unit limited to named, trained personnel.<br>Leave clothes inside before shower. Full shower on exit from unit.<br>Personnel not permitted to visit premises with pigs for 48 hours after leaving unit.
Equipment	All equipment required for animal procedures to be available within the unit. All materials to be sterilised on removal from unit or, in the case of animal samples, to be double-wrapped in leakproof container which is surface disinfected for transport to the ASF laboratory.
Animals	All animals to be slaughtered before leaving the unit, post-mortem examinations to be completed within the bio-safe area, and carcases incinerated on completion of examinations.
